# Bioprocess development for biosurfactant production by *Natrialba* sp. M6 with effective direct virucidal and anti-replicative potential against HCV and HSV

**DOI:** 10.1038/s41598-022-20091-0

**Published:** 2022-10-04

**Authors:** Ghada E. Hegazy, Marwa M. Abu-Serie, G. M. Abou-elela, Hanan Ghozlan, Soraya A. Sabry, Nadia A. Soliman, Mohamed Teleb, Yasser R. Abdel-Fattah

**Affiliations:** 1 National Institute of Oceanography & Fisheries, NIOF-Egypt, Qaitbay Sq, El-Anfousy, Alexandria, 11865 Egypt; 2 Medical Biotechnology Department, Genetic Engineering & Biotechnology Research Institute (GEBRI), City of Scientific Research & Technological Applications, Alexandria, Egypt; 3 Botany & Microbiology Department, Faculty of Science, Alexandria University, Alexandria, Egypt; 4 Bioprocess Development Department, Genetic Engineering & Biotechnology Research Institute (GEBRI), City of Scientific Research & Technological Applications, New Borg El-Arab City, Universities & Research Institutes Zone, Alexandria, 21934 Egypt; 5 Department of Pharmaceutical Chemistry, Faculty of Pharmacy, Alexandria University, Alexandria, Egypt

**Keywords:** Biotechnology, Microbiology

## Abstract

Halophilic archaea is considered an promising natural source of many important metabolites. This study focused on one of the surface-active biomolecules named biosurfactants produced by haloarchaeon *Natrialba* sp. M6. The production trend was optimized and the product was partially purified and identified using GC–Mass spectrometry. Sequential optimization approaches, Plackett–Burman (PB) and Box–Behnken Designs (BBD) were applied to maximize the biosurfactants production from M6 strain by using 14 factors; pH, NaCl, agitation and glycerol; the most significant factors that influenced the biosurfactant production were used for Response Surface Methodology (RSM). The final optimal production conditions were agitation (150 rpm), glycerol (3%), NaCl (20.8%), pH (12) and cultivation temperature (37°C). GC–Mass spectrometry for the recovered extract revealed the presence of a diverse group of bipolar nature, hydrophobic hydrocarbon chain and charged function group. The majority of these compounds are fatty acids. Based on results of GC–MS, compositional analysis content and Zetasizer, it was proposed that the extracted biosurfactant produced by haloarchaeon *Natrialba* sp. M6 could be a cationic lipoprotein. The antiviral activity of such biosurfactant was investigated against hepatitis C (HCV) and herpes simplex (HSV1) viruses at its maximum safe doses (20 μg/mL and 8 μg/mL, respectively). Its mode of antiviral action was declared to be primarily via deactivating viral envelopes thus preventing viral entry. Moreover, this biosurfactant inhibited RNA polymerase- and DNA polymerase-mediated viral replication at IC_50_ of 2.28 and 4.39 μg/mL, respectively also. Molecular docking studies showed that surfactin resided well and was bound to the specified motif with low and accepted binding energies (ΔG = − 5.629, − 6.997 kcal/mol) respectively. Therefore, such biosurfactant could be presented as a natural safe and effective novel antiviral agent.

## Introduction

Biosurfactants is a different group of surface-active biomolecules produced by several living organisms^1^, with amphiphilic compounds consisting of hydrophobic and hydrophilic tails^2^. Biosurfactants were first discovered as extracellular compounds through studying the hydrocarbons fermentation. They received attention as “alternative surfactants” since they have several advantages greater than synthetic surfactants. These advantages include good biodegradability, low toxicity to mammalian cells and tissues, selectivity, low irritancy, effectiveness at extreme conditions of temperatures or pH values, suitability for large-scale production and ecological acceptability^3^. Nowadays, biosurfactants are used in various industries such as a cosmetic, agriculture, pharmaceutics and oil-contaminated environments bioremediation^2^. Biosurfactants are usually classified based on the microorganisms producing them and the nature of their chemical structures. Major biosurfactant classes include phospholipids, lipoprotein, fatty acids, glycolipids, and polymeric surfactants. Most of these compounds are either anionic or non anionic. Only a few of them are cationic, for example those containing amine groups. Biosurfactant-producing organisms are very different and come from various environments, including soil, seawater, marine sediments, oil contaminated soil, fields of the oil and even extreme habitats^4^. The search for new biosurfactant compounds in extremophiles organisms appears to be mostly promising, since they have unique adaptations to harsh environments^4^. In spite of the few studies on biosurfactant-producing organisms in hypersaline habitats, there has been a greater increase in interest in halophilic archaea and bacteria for biosurfactant production in recent years. Because of the difficulty in removing and degrading complex hydrocarbon-compounds that have contaminated hypersaline environments, using halophilic microorganisms as archaea is both environmentally and cost-effective. Microorganisms which live and adapted in harsh environments, play an important role in the bioremediation process by using organic pollutants as the sole carbon source for the production of biosurfactants^5^. Kebbouche-Gana et al. isolated and characterized halophilic archaea capable of producing biosurfactants for hydrocarbon degradation^6^. Halophilic archaea composed of unique lipid (phytanylglycerol), which may have a principle role to act as surface active agent. PB and BB statistical techniques were used to reach the optimum conditions for the biosurfactants production^7^. Viruses are responsible for several serious outbreaks and pandemics all over the world^8^. Genetic material of virus is encased by protein layer of capsid (non-envelope virus), whereas virion, in envelope virus, the capsid is enclosed by lipid bilayer that contains viral proteins that facilitate the viral binding to the host cells. Envelope viruses mainly transmit through body fluids (e.g., hepatitis viruses, herpes simplex virus (HSV), and human immunodeficiency virus type 1), and respiratory route (e.g., SARS-CoV2). Thus, envelope viruses pose a significant health risk, and are considered the causative agents of pandemics and major disease outbreaks^9^. In this context, biosurfactant exhibited higher viral inactivation against envelope viruses than non-envelope viruses. This is primarily attributable to the physicochemical properties of biosurfactant, which mediate interaction with the hydrophobic domain of the lipid membrane of envelope viruses, causing it to be disrupted^10^. Researchers are focusing on the discovering of safe and effective broad-spectrum antiviral agents^8^. Because of viral chemoresistance, the direct virucidal^10^. Effect of biosurfactant is a key factor in the developing alternative therapeutic effective agent Moreover, recent study demonstrated that biosurfactant had immunomodulatory activity with anti-inflammatory potential^11^. Accordingly, the present study optimized, for the first time, the production of biosurfactants by an extreme halophilic archaeon strain M6, as well as its antiviral activity against hepatitis C virus and HSV1 was investigated. HCV is the main cause of liver cirrhosis and cancer. HCV has been treated with interferon-free direct-acting antivirals (IFN-free DAAs) since 2002, despite their numerous side effects^12^. Sofosbuvir is the most recently used IFN-free DAA. Despite the availability of effective anti-HCV treatments, 71 million people were infected with HCV in 2019. This is because the number of new infections has been nearly equal to the number of successful treatments^13^. Thus, in addition to viral replication inhibitors, a safe blocker agent of HCV entry will be required. HCV contains a positive single strand RNA which is associated with core protein and enveloped by two glycoproteins (E1 and E2). These latter proteins are responsible for viral entry (the initial phase of HCV life cycle). E2 has been studied extensively as envelope protein-mediated receptor binding and is the main target of neutralizing antibodies^14,15^. Following viral entry, the incoming HCV genome is translated into a single polypeptide which is then processed by host and viral proteases generating structural and non-structural proteins (NS). For HCV replication, the most important NS protein is RNA-dependent RNA polymerase (NS5B)^16^. Dual targeting E2 and NS3 is therefore critical in the development of effective anti-HCV agents. Accordingly, the antiviral activity of the extracted biosurfactant was investigated in this study by assessing its inhibitory potency on these two key mediators (E2 and NS5B) of HCV life cycle. Herpes simplex viruses causes recurrent mucosal lesions in oral and genital area and can infect central nervous system. HSV-1 is the most common cause of oral and corneal lesions, as well as adult encephalitis, whereas HSV-2 is more commonly associated with genital herpes and meningitis^16^. HSV entry begins with attachment of its glycoproteins to the cell surface followed by interactions between HSV glycoprotein D (gp D) and cellular receptors to facilitate capsid penetration and viral replication^17^. There are many commercially available anti-HSV drugs (nucleoside analogs) which primarily work by interfering with the function of HSV DNA polymerase. As a result, mutant HSV variants may be resistant to these drugs, resulting in encephalitis and blindness, especially in immunocompromised patients^18^. Hence, the purpose of this study is to first optimizing the production and characterizing the extracted biosurfactant from haloarchaeon *Natrialba* sp. and then to investigate the modes of its antiviral activity against HCV and HSV1 (acyclovir-sensitive strain “KOS”). More importantly, its major antiviral modes (direct virucidal and anti-replicative potential) were thoroughly evaluated against key mediators of HCV (E2 and NS5B) and HSV1 (gp D and polymerase) and computational molecular docking studies was done to show that surfactin resided well and was bound to the specified motif with low binding energies.

## Results

### Chemical analysis of water and sediment samples

Water and sediments samples were collected from Egyptian saline Lake Wadi El-Natrun (El-Hamra Lake), Egypt. Wadi El-Natrun is located at 80 km northwest of Cairo; 23 m below sea level. The salinity and alkalinity of El-Hamra Lake reach to 4.5 M and pH 11^19^. Data of chemical analysis for water and sediments samples of El-Hamra Lake, Wadi El-Natrun are presented in Table 1, including potassium, magnesium, calcium, silica, phosphate, chlorine, sulfate, CO_3_, HCO_3_ and total nitrogen during April and September 2013. The data revealed that the main ions contributing to the salinity of the lake were chloride, carbonate, sulfate and potassium which represent about 99% of the total salinity. The predominant cation was potassium in detectable amounts ranging from 28.96 to 31.49 mg/L and chloride as anion ranging from 20,990 to 56,100 mg/L. High concentrations of sulfate (114,697–115,303 mg/L), phosphate (1202–1214 mg/L), and carbonate (900–3000 mg/L) were detected. The heavy metals ions were below the detection limit. In contrast the biologically important compounds such as carbonate, phosphate, and sulfate were present in large amounts^19,20^.

**Table 1:** Water and sediment samples analysis.

	April/2013	September/2013
**Water parameters (mg/L)**		
Potassium	28.96	31.49
Magnesium	9.040	9.000
Calcium	2.850	1.170
Silica	270.0	270.90
Phosphate	1214.0	1202.0
Chloride	20,990	56,100
Sulfate	114,697	115,303
CO_3_	3000.0	900.00
Total nitrogen	34.72	61.600
Total ALKALINITY (HCO_3_)	259,200	242,000
**Sediment parameter (mg/g)**		
Potassium	3.100	2.050
Magnesium	0.440	2.120
Calcium	0.96	1.900

Triplet tests were performed and the average of three readings was considered as the final.

### Screening for biosurfactant production

**Figure 1:**
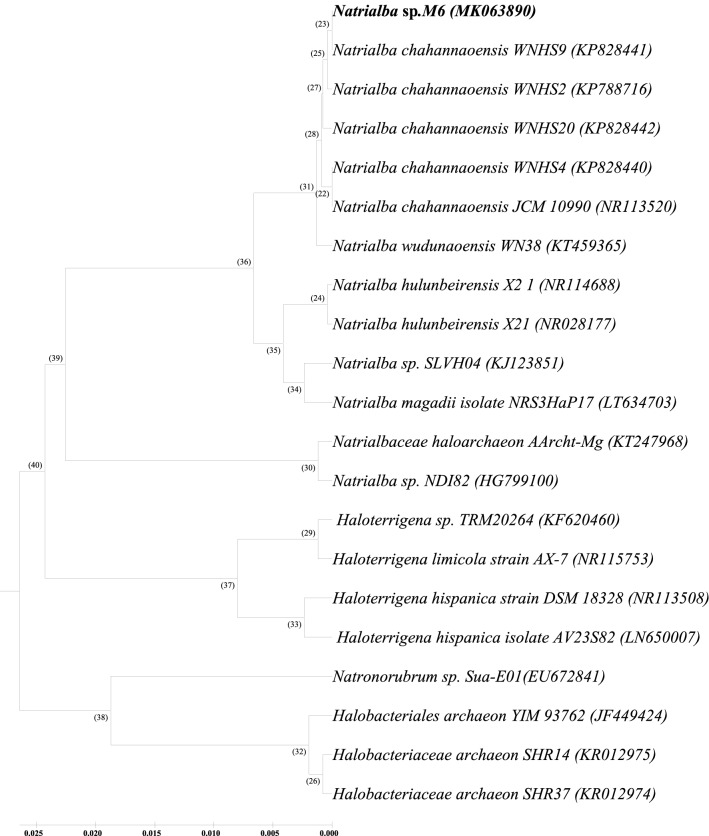
A Phylogenetic tree, based on the 16S *rRNA* gene sequence comparison showing the position of the haloalkaliphilic archaeal isolate coded M6, from El Hamra Lake, Wadi El Natrun, and its closest relatives.

Among 10 tested archaeal isolates that showed positive to oil displacement method (one of the test used for detection of the biosurfactant activity), only one isolate, coded M6, was selected to complete this study. Data in Table 2 demonstrated that M6 is considered a promising and an effective biosurfactant-producer among all tested isolates as indicated by the highest (60%) Emulsification index percentage (EI%)_,_ the most reduction in the surface tension (ST) and a clear zone appearance in blood haemolytic test.Worth to mention, the selected strain was previously, isolated from El-Hamra Lake, Wadi El-Natrun, sediment), identified as *Natrialba* sp. M6, kept in GenBank under accession number (MK063890) and known as a producer of carotenoids^21^ Based on formerly submitted 16s *rRNA* sequence result (ac: Mk063890), a phylogenetic tree was displayed and found M6 is very close to the cluster of *Natrialba chahannaoensis* strains (Fig. 1) and the nearest is WNHS9 strain with an identity % 99.63.

**Table 2:** Screening of biosurfactant-producing haloarchaea isolates.

Isolates	Oil displacement test	ST (mN/m)	(EI_24%_)	Haemolytic test
M6	+	45.0	1.5/2.5 (60%)	+
A8	+	47.6	–	−
A3	+	48.8	–	−
RA1	+	46.9	–	−
A5	+	46.2	1.3/2.5 (52%)	+
RE5	+	47.9	–	−
M7	+	46.6	–	−
RA2	+	54.7	–	−
GH6	+	54.5	–	−
S8	+	47.3	–	−
− ve control	−	65.0	–	−
+ ve control	+	33	1.6/2.5 (64%)	+

Triplet tests were performed and the average of three readings was considered as the final.

− ve control is the medium while the + ve control is Tween20 at 1%.

### Optimization of *Natrialba* sp. M6 surfactant production using experimental designs

For this screening step, 14 variables at two different levels, high and low were selected to evaluate biosurfactant production. Table 3 presents the design template and the individual response results namely: actual, predicted & residual as the reciprocal of the corrected ST × 1000 of the different trials. Also the real levels for coded values (1, 0, − 1) of the studied variables were represented in Table 3. The main effect of each variable on the ST was estimated as the difference between the average of the measurements at the high (1) and low (− 1) levels of that factor.

**Table 3:** PB experimental design for the evaluation of the factors influencing biosurfactant activity of *Natrialba* sp. M6.

Trials	Variables														Response*		
	X1	X2	X3	X4	X5	X6	X7	X8	X9	X10	X11	X12	X13	X14	Actual	Predicted	Residual
1	− 1	1	1	1	1	− 1	1	− 1	1	1	1	− 1	1	− 1	17.699	18.18278	− 0.48366
2	1	− 1	− 1	− 1	1	− 1	− 1	− 1	− 1	1	1	− 1	1	1	17.889	17.40542	0.483663
3	− 1	− 1	− 1	− 1	1	1	1	1	1	− 1	− 1	− 1	1	1	16.155	16.63875	− 0.48366
4	1	1	− 1	− 1	1	1	1	− 1	1	− 1	1	1	− 1	− 1	17.271	16.78749	0.483663
5	1	− 1	1	1	1	1	− 1	1	1	1	− 1	1	1	− 1	17.183	16.69847	0.483663
6	− 1	− 1	1	1	1	1	− 1	− 1	− 1	− 1	1	1	− 1	1	17.301	17.7847	− 0.48366
7	1	− 1	− 1	1	− 1	1	1	− 1	− 1	1	− 1	− 1	− 1	− 1	16.863	17.34707	− 0.48366
8	1	1	− 1	1	− 1	− 1	− 1	− 1	1	− 1	− 1	1	1	1	16.077	16.56083	− 0.48366
9	1	1	1	1	1	− 1	1	1	− 1	− 1	− 1	− 1	− 1	1	22.936	22.45212	0.483663
10	1	− 1	1	− 1	− 1	− 1	− 1	1	1	− 1	1	− 1	− 1	− 1	15.479	15.96354	− 0.48366
11	1	1	1	− 1	− 1	1	1	1	− − 1	1	1	1	1	1	15.337	15.82109	− 0.48366
12	− 1	− 1	1	− 1	− 1	− 1	1	− 1	− 1	− 1	− 1	1	1	− 1	17.271	16.78749	0.483663
13	− 1	1	− 1	1	− 1	1	− 1	1	− 1	− 1	1	− 1	1	− 1	17.064	16.58118	0.483663
14	− 1	1	− 1	− 1	1	− 1	− 1	1	− 1	1	− 1	1	− 1	− 1	17.452	17.93567	− 0.48366
15	− 1	1	1	− 1	− 1	1	− 1	− 1	1	1	− 1	− 1	− 1	1	15.360	14.87732	0.483663
16	− 1	− 1	− 1	1	− 1	− 1	1	1	1	1	1	1	− 1	1	15.128	14.64493	0.483663

Variables				Code	Coded level and actual level												
					− 1									+ 1			
Temperature (°C)				X1	35									40			
pH				X2	8									10			
Casamino acids (g%)				X3	0.5									1.0			
NaCl (g%)				X4	10									20			
Agitation (rpm)				X5	0									200			
Glucose (g%)				X6	0.1									1.0			
Glycerol (g%)				X7	0.1									1.0			
NH4Cl (g%)				X8	0.01									0.1			
NH4NO3 (g%)				X9	0.01									0.1			
Yeast extract (g%)				X10	0.1									1.0			
(NH4)_2_SO4 (g%)				X11	0.01									0.1			
MgSO4·7H_2_O (g%)				X12	0.01									0.1			
CaCl2 (g%)				X13	0.0									0.1			
FeSO4·7H_2_O (g%)				X14	0.0									0.0001			

Triple tests were performed and the average of three reading was considered as the final.

*Response is the reciprocal of the measured ST × 1000.

### Statistical analysis of the PBD

PBD is two levels experimental design; it involves a linear polynomial correlation model that describes the correlation between the 14 factors and the response as follow:

$${\text{Y}} = { 16}.{9}0{7 } + \, 0.{\text{35 X}}_{{1}} + \, 0.{\text{371 X}}_{{2}} + \, 0.{\text{292 X}}_{{3}} + \, 0.{5}0{\text{2 X}}_{{4}} + \, 0.{\text{956 X}}_{{5}} - \, 0.{\text{46 X}}_{{6}} + \, 0.{3}0{\text{3 X}}_{{7}} + \, 0.0{\text{62 X}}_{{8}} - \, 0.{\text{74 X}}_{{9}} - \, 0.{\text{42 X}}_{{{1}0}} - \, 0.{\text{38 X}}_{{{11}}} - \, 0.{\text{4 X}}_{{{12}}} - \, 0.{\text{19 X}}_{{{13}}} - 0.00{\text{515 X}}_{{{14}}} .$$

**Figure 2:**
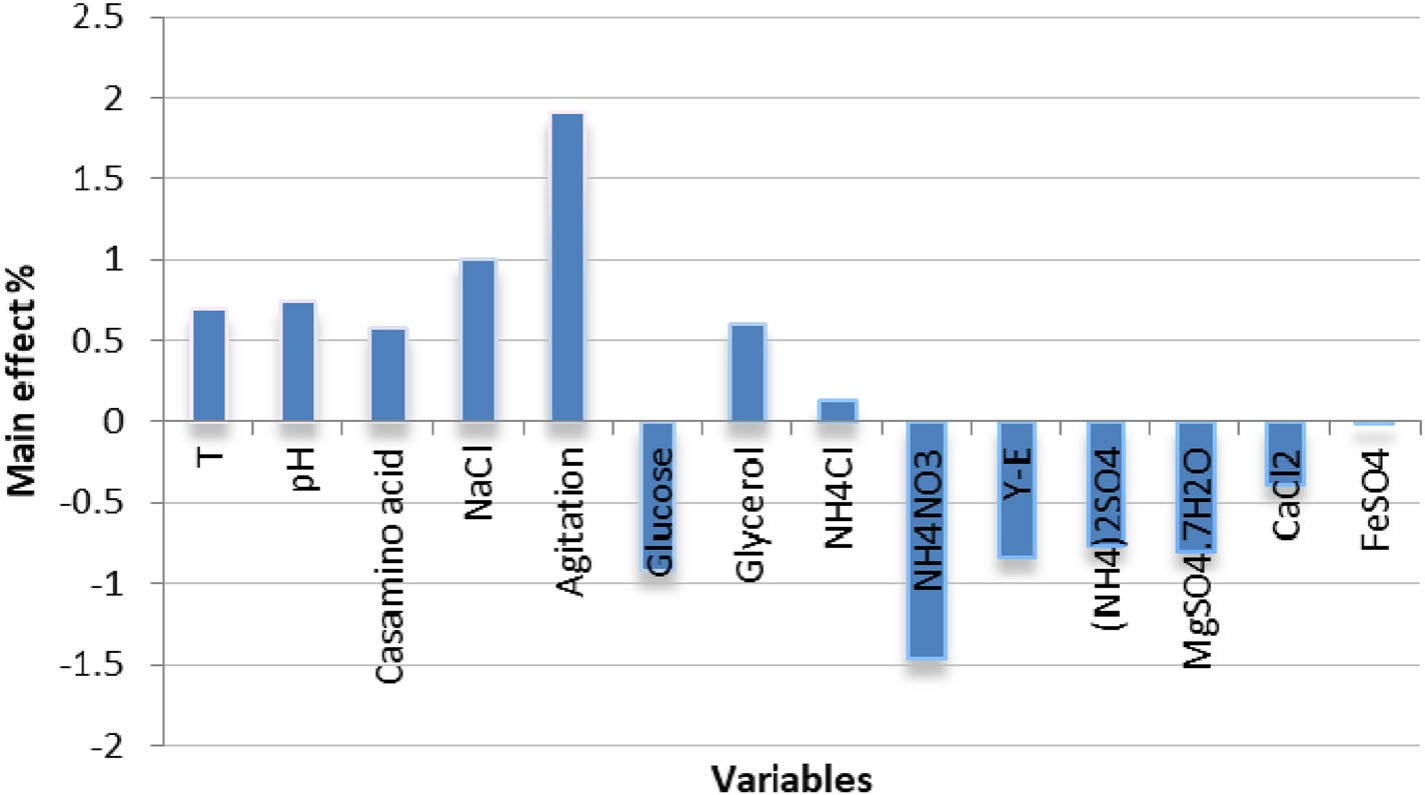
Main effect of the different factors influencing the biosurfactant production by *Natrialba* sp. M6 based on PBD.

Variance analysis for data was carried out using the ANOVA method using MICROSOFT EXCEL tools and JMP-program. The *R*2 value is considered a good measurable for a model quality. In this work the obtained value (0.92488) for *R*2 indicates that the predicted model is highly fit and explains more than 92% of biosurfactants variation. Based on main effect results shown in Fig. 2, the most significant factors affecting biosurfactant production are agitation, ammonium nitrate, sodium chloride, glucose, yeast extract, MgSO_4_·7H_2_O, NH_4_SO_4_, pH, T, glycerol, casamino acid, CaCl_2_, NH_4_Cl and FeSO4·7H2O in descending order. By analyzing the regression coefficient for the 14 variables, it is concluded that pH, agitation, glycerol, temperature, casamino acids, NH_4_Cl and NaCl showed positive effect on the biosurfactant activity. On the other hand, all other variables showed negative effect on biosurfactant production. Therefore, pH, glycerol, agitation and sodium chloride were selected as significant factors for further optimization. Pre-optimization experiment was carried out by using the high level of the most significant variables and the low level of the other variables.

### Optimization of the culture conditions using BBD

To identify the optimum response region for biosurfactant production, the significant independent variables (pH, X_1_; glycerol, X_2_; agitation, X_3_; sodium chloride, X_4_) were further explored at three levels. Table 4 presents the design pattern for the selected variables, the response results (actual, predicted & residual) of each trial, where the response here was the reciprocal of the corrected ST × 1000 (1/ST × 1000). Also, the real levels for coded values (1, 0, − 1) of the selected variables were presented in Table 4 as well. To predict the optimal point, a second order polynomial function was fitted to the experimental response results (non-linear optimization algorithm).

$${\text{Y}} = {22}.{69136 } + { 2}.{311}0{\text{71X}}_{{1}} + { 1}.{\text{297846X}}_{{2}} - { 3}.{4}0{\text{958X}}_{{3}} + \, 0.{1917}0{\text{5X}}_{{4}} + \, 0.{\text{661656X}}_{{1}} {\text{X}}_{{2}} - { 6}.{7}0{8}0{\text{2X}}_{{1}} {\text{X}}_{{3}} + \, 0.{\text{324812X}}_{{1}} {\text{X}}_{{4}} - {8}.{\text{66472X}}_{{2}} {\text{X}}_{{3}} + 0.{\text{237175X}}_{{2}} {\text{X}}_{{4}} - \, 0.{\text{22231X}}_{{3}} {\text{X}}_{{4}} + \, 0.{49}0{\text{423X}}_{{1}}^{{2}} - \, 0.{\text{84625X}}_{{2}}^{{2}} + {4}.{732}0{\text{54X}}_{{3}}^{{2}} - {3}.00{\text{752X}}_{{4}}^{{2}} .$$

**Table 4:** BB factorial experimental design for biosurfactant production by *Natrialba* sp. M6.

Trials	Variables				Response*		
	X1	X2	X3	X4	Actual	Predicted	Residual
1	0	0	0	0	20.69	20.08573	0.605625
2	0	0	1	− 1	20.37	21.03691	− 0.67031
3	− 1	− 1	0	0	22.52	19.38827	3.134256
4	0	0	1	1	19.92	20.97571	− 1.05539
5	0	0	− 1	1	23.75	28.23948	− 4.48651
6	1	− 1	0	0	17.70	22.6871	− 4.98798
7	0	0	− 1	− 1	23.31	27.41146	− 4.10144
8	− 1	1	0	0	20.49	20.66065	− 0.16884
9	1	1	0	0	18.32	26.6061	− 8.29108
10	0	0	0	0	19.12	20.08573	− 0.96979
11	0	1	1	0	20.70	15.8007	4.903232
12	0	− 1	1	0	31.35	30.53446	0.813507
13	0	1	− 1	0	42.92	39.94931	2.969148
14	1	0	0	− 1	21.93	21.96881	− 0.03899
15	− 1	0	0	1	21.55	17.73008	3.821643
16	0	− 1	− 1	0	18.90	20.02417	− 1.12058
17	− 1	0	0	− 1	20.45	17.9963	2.453602
18	1	0	0	1	24.33	23.00185	1.329053
19	0	− 1	0	− 1	19.16	17.58521	1.571881
20	0	1	0	1	20.37	20.56431	− 0.19771
21	0	1	0	− 1	20.50	19.70655	0.785255
22	− 1	0	− 1	0	20.37	22.30432	− 1.93773
23	0	0	0	0	20.45	20.08573	0.364165
24	1	0	1	0	23.42	20.10731	3.311897
25	1	0	− 1	0	49.02	40.34251	8.677103
26	0	− 1	0	1	18.08	17.49427	0.588916
27	− 1	0	1	0	21.60	28.9012	− 7.30293

Variables	Code	Coded level and actual level					
		− 1	0	+ 1			
pH	X1	10	11	12			
Glycerol (g%)	X2	1	2	3			
Agitation (rpm) NC	X3	150	200	250			
NaCl (g%)	X4	15	20	25			

Triple tests were performed and the average of three reading was considered as the final.

*Response is the reciprocal of the measured ST × 1000.

**Figure 3:**
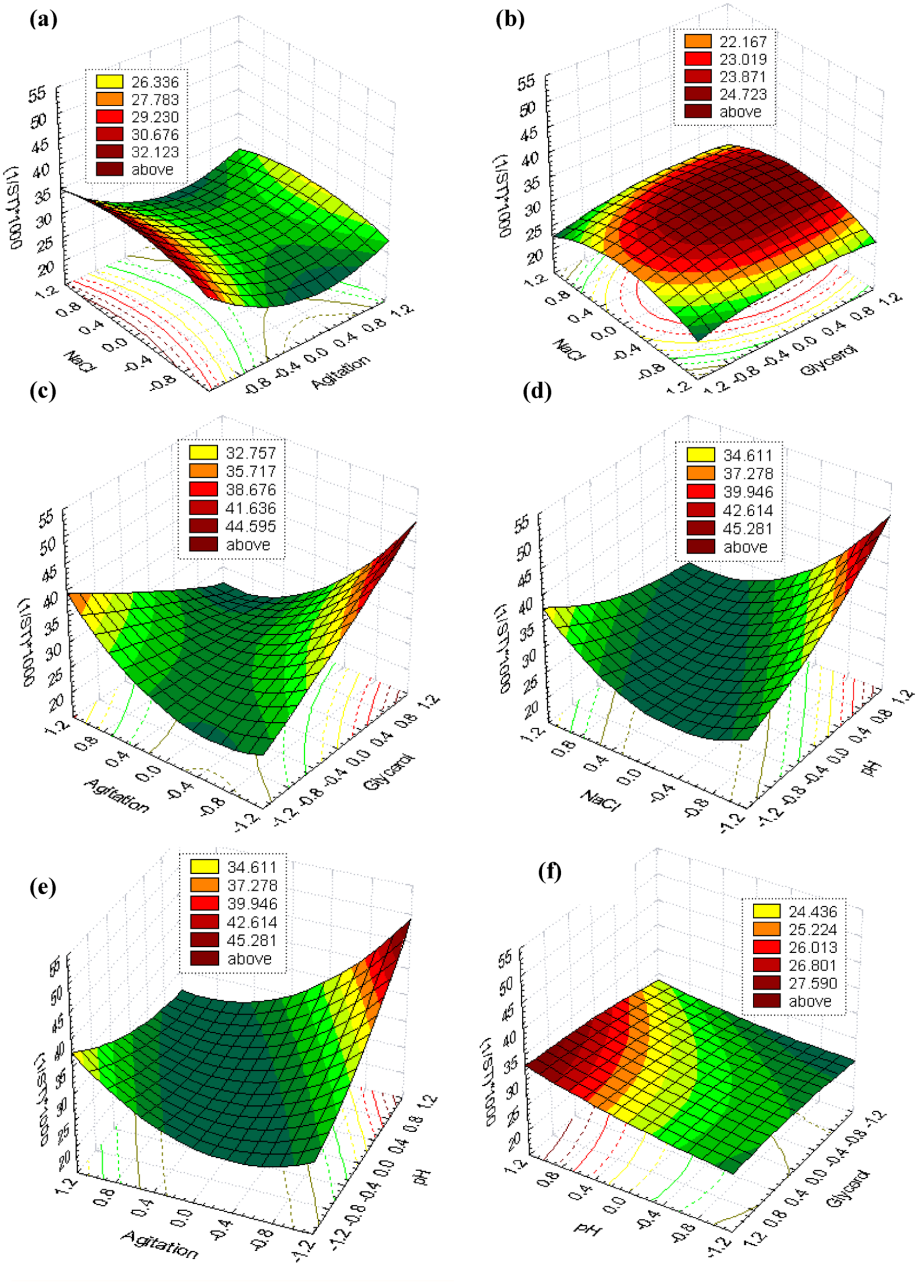
Three-dimensional surface and contour plots showing the relationships between the tested variables and the biosurfactant as a response in a form of [(1/ST) × 1000] produced by *Natrialba* sp. M6. (**a**) Showed that at low agitation value and middle value of NaCl gave the highest biosurfactant production level. (**b**) Showed that at high glycerol value and middle value of NaCl gave the highest biosurfactant production level. (**c**) Showed foci for maximum level of biosurfactant production at high glycerol value and low agitation. (**d**) Indicated that at middle NaCl and high pH values the maximum production was achieved. (**e**) Showed the maximum response at high pH and low agitation and (**f**) showed foci for maximum level of biosurfactant production at high values of both glycerol and pH.

**Figure 4:**
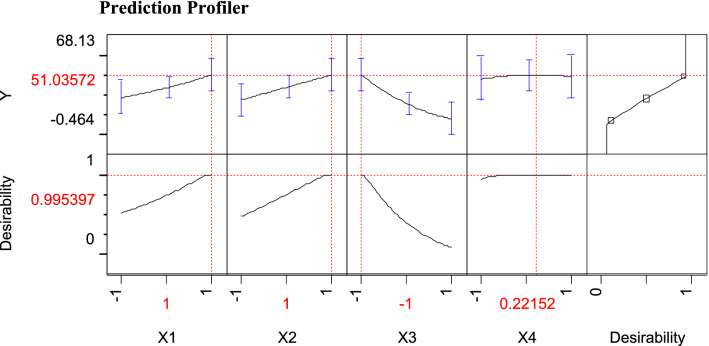
JMP Desirability prediction profile showing the predicted optimal levels of studied four variables, X1 (pH), X2 (Glycerol), X3 (agitation) and X4 (NaCl); along with the predicted biosurfactant as (1/ST) × 1000 produced by *Natrialba* sp. M6.

On the model level, the correlation measures for estimating the regression equation are the multiple correlation coefficients *R* and the determination coefficient *R*^2^. In this experiment, the value of *R*^2^ was 0.837 for the biosurfactant production, indicating a great degree of correlation between the experimental and the predicted values. Figure 3 shows the simultaneous effects of the four most significant independent factors on each response using three-dimensional graphs generated by STATISTICA 7.0 software. Additionally, a desirability profile shows the optimal levels of the four studied variables as obtained from the maximum point of the polynomial model were estimated using the *SOLVER* function of MICROSOFT EXCEL tools and JMP-program, and found to be: pH 12 (1), glycerol 30 g/L (1), agitation 150 rpm (− 1) and NaCl 200.8 g/L (0.2215), with a predicted response of 51.03 (Fig. 4).

### Identification of partially purified *Natrialba* sp. M6 biosurfactant

**Figure 5:**
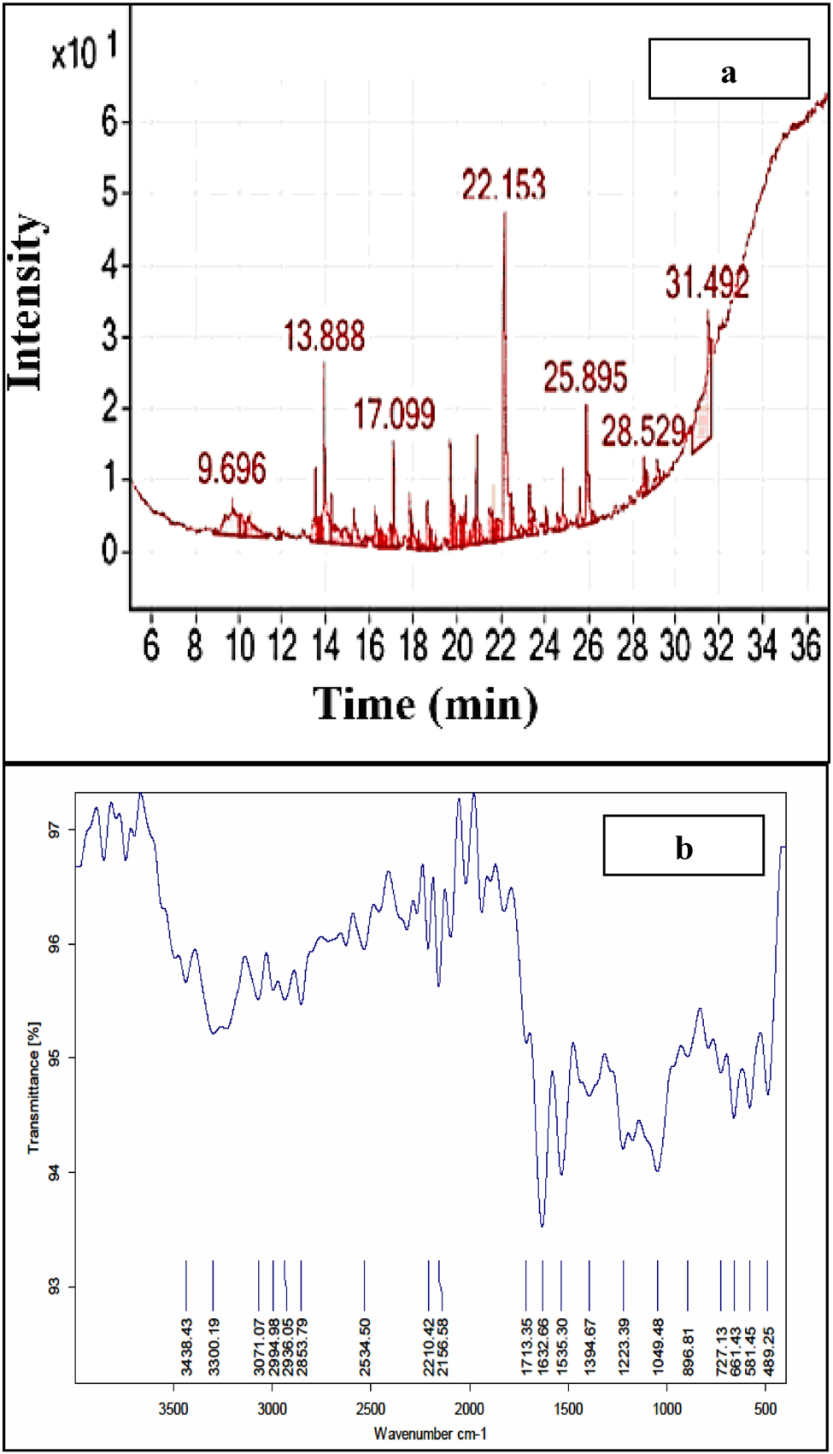
Chemical analysis of the partially purified extracted biosurfactant. (**a**) Gas chromatography–mass spectrometry (GC–MS) analysis. (**b**) Fourier transform infrared (FTIR) spectroscopy analysis.

The GC–MS analysis of the partially extracted biosurfactant, as shown in Fig. 5a, indicates the presence of different biosurfactant components. It also provided a detailed description of the existing major bands. The presented data revealed that the recovered extracts contained a diverse group consisting of 34 compounds, the majority of which were bipolar nature, hydrophobic hydrocarbon chain and charged function group; additionally, the most of these compounds are fatty acids. The major biosurfactant-related components that could be characterized were octadecane,1-[2-(hexadecyloxy)ethoxy] RT (9.696), ethanol, 2-(octadecyloxy) RT (13.525), phenol 2,4-bis(1,1-dimethylethyl) RT (13.894), 13-dioxane,5-(hexadecyloxy)-2-pentadecyl-, trans RT (15.292), ethyl iso-allocholate RT (16.245), tert-hexadecanethiol RT (18.641), pentadecanoic acid, ethyl ester RT (19.716), and 14-hydroxy-15-methylhexadec-15-enoic acid, ethyl ester RT (21.500), as identified by gas chromatography mass spectrophotometer (GC–MS) analysis. Also, Fourier transform infrared (FTIR) spectroscopy was carried out to illustrate the chemical structure of the partially purified biosurfactant of *Natrialba* sp. M6. The FTIR spectroscopic analyses indicated the presence of various chemical groups in the extracted biosurfactant, as shown in Fig. 5b. The bands appearing at (489.25, 581.45, 661.43 and 727.13) cm^−1^ correspond to alkyl halides compounds C–I and C–Cl, respectively. The band at 896.81 is assigned to C–H bending. A band at 1049.48 cm^−1^ correspond to polysaccharides was also detected. The bands at (1394.67 to1535.30 and 1632.66 to 1713.33) cm^−1^ is matching to C=O and C–O stretching, respectively. Meanwhile, band appearing at (2534.5, 2853.79 3300.19 and 3438.43) cm^−1^ correspond to S–H, C–H, N–H and O–H stretching of amines and amides respectively. The characteristic stretching frequency of amides may be attributed to histidine present in biosurfactant.

### Percentage composition, elementals (dry weight) of biosurfactant sample and its surface charge

The concentrations of protein, carbohydrate, and lipid contents of *Natrialba* sp. M6 biosurfactant were found to be 31.599, 11.72, and 41.53 mg/g, respectively which showed that the majority of its components are lipids. The percentages of elementals C, N, H and S of *Natrialba* sp. M6 biosurfactant were found 13.77, 3.19, 2.106, 0.151%, respectively which showed that C:N ratio (4.3) & C:H ratio 6.5. Zetasizer illustrated that biosurfactant extract have a low positive charge (0.559 mV).

### Anti-HCV and anti-HSV1 activity of biosurfactant with disclosing its mode of action

#### Cytotoxicity doses on viral host cells and effective antiviral doses with selectivity index of biosurfactant

Before investigating the isolated biosurfactant’s antiviral efficacy, its cytotoxicity on viability of host cells (peripheral blood mononuclear cells (PBMCs) and Vero cell line) of HCV and acyclovir sensitive-HSV1, respectively, should be assessed using MTT assay. Cytotoxicity results (Supplementary Fig. 1) of this experiment illustrated that safe doses of biosurfactant, at which 100% viability of normal cells (PBMCs and Vero), were 20.41 ± 1.71 μg/mL and 8.17 ± 0.31 μg/mL, respectively. The safe doses of standard anti-HCV and anti-HSV1 drugs (sofosbuvir and acyclovir, respectively) were 188.33 ± 13 μg/mL and 266.12 ± 5.1 μg/mL, respectively. The estimated cytotoxicity concentrations at 50% cell viability (CC_50_) of biosurfactant for PBMCs and Vero cells were 326.3 ± 1.8 μg/mL and 268.2 ± 5.9 μg/mL, respectively and for sofosbuvir and acyclovir were 1114 ± 11 μg/mL and 1234 ± 5.1 μg/mL, respectively (Supplementary Fig. 1). MTT assay was used to estimate TCID50 of HSV1 (10^–4^) that cause 50% cell lysis. Moreover, this assay was used to detect the inhibitory potency of biosurfactant and standard drugs in HCV-infected PBMC and on standard infectious dose (100 TCIC50) of HSV1-mediated Vero lysis at serial concentrations of the above-mentioned safe dose (EC_100_ = 20 and 8 μg/mL, respectively). After 72 h of biosurfactant treatment for viral infected cells, the doses (EC_50_) of biosurfactant that required to eliminate HCV by 50% and inhibit virus-mediated cell lysis by 50% were calculated. The recorded EC_50_ values for anti-HCV activity of biosurfactant and sofosbuvir were 20.12 ± 1.8 μg/mL and 9.81 ± 0.2 μg/mL, respectively, and for anti-HSV1 activity of biosurfactant and acyclovir were 8.569 ± 0.2 μg/mL and 5.968 ± 0.2 μg/mL, respectively (Supplementary Fig. 1). Furthermore, the selectivity index (SI; ratio of CC_50_ to EC_50_) was estimated, it was found that SI values for anti-HCV potential of biosurfactant and sofosbuvir were 16.33 ± 1.3 μg/mL and 113.7 ± 3.1 μg/mL, respectively, and for anti-HSV1 potency of biosurfactant and acyclovir were 31.33 ± 1.4 μg/mL and 207.2 ± 11 μg/mL, respectively (Supplementary Fig. 1).

#### Potential direct virucidal and anti-replicative as major antiviral modes of biosurfactant

**Figure 6:**
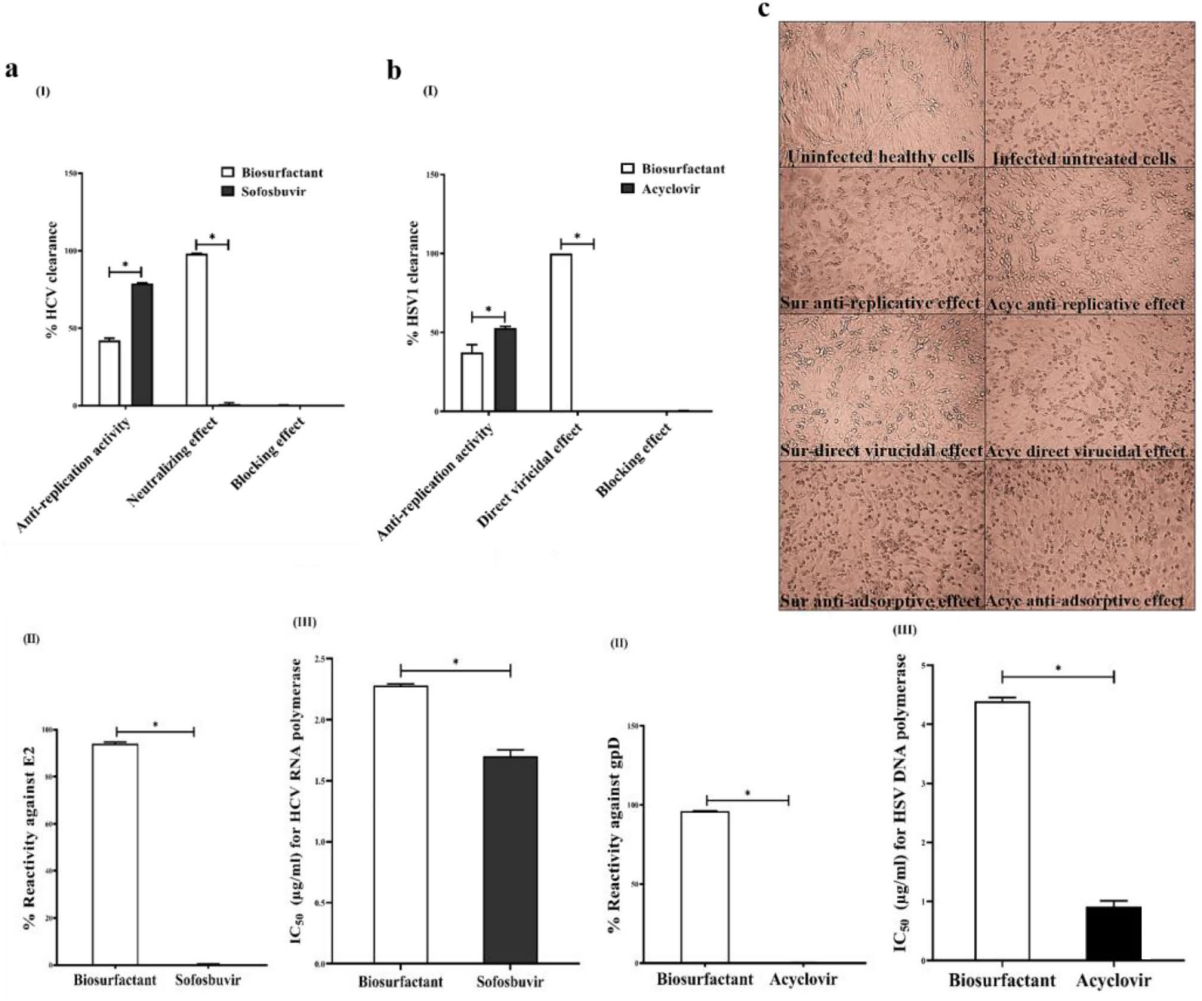
Antiviral activity of biosurfactant with unveiling its mechanism(s)-mediated antiviral mode, compared to standard drugs against (**a**) HCV and (**b**) HSV1. (**I**) QPCR-estimated HCV and HSV1 eliminating percentage, relative to the untreated infected cells, in three suggested modes of antiviral action, including anti-replicative, neutralizing and blocking effects at safe concentration (EC_100_) of biosurfactant, (**II**) the relative reactivity % of biosurfactant against HCV-envelope protein (E2) and HSV-glycoprotein (gp) D and (**III**) IC_50_ values of biosurfactant for inhibiting HCV RNA polymerase and HSV DNA polymerase. All data are expressed as mean ± SEM and considered significantly different at p < 0.05*.

The anti-HCV and anti-HSV1 activity of biosurfactant comparing with currently used drugs, at EC_100_ (20 μg/mL and 8 μg/mL, respectively), was investigated by three protocols. The tested biosurfactant was incubated with viruses-infected cells, preincubated with viruses before added to host cells or preincubated with host cells before adding viruses to cells for testing its anti-replicative, neutralizing/direct virucidal or blocking action of antiviral effect, respectively (Fig. 6aI). The intracellular HCV and HSV1 were quantified using qPCR. As shown in Fig. 6aI, biosurfactant can eliminate 98.09 ± 0.213% of HCV, relative to the untreated infected PBMCs, when directly preincubated with virus but it exhibited a lower viral clearing potential (42.12 ± 1.45%) in the case of its addition on HCV-infected cells. HCV load did not differ significantly between the untreated HCV-infected PBMCs and those that had been preincubated with biosurfactant before being exposure to HCV (Fig. 6aI). Also, 8 μg/mL of biosurfactant exhibited perfect virucidal activity (99.98%) when was preincubated with HSV1 rather than its anti-replicative effect (37.41%) which was investigated by adding to HSV1-infected Vero cells. Meanwhile, no discernible anti-adsorptive HSV1 activity was recorded for biosurfactant that was preincubated Vero cells before infection (Fig. 6bI). This indicates that antiviral activity of biosurfactant is mainly by its neutralizing or direct virucidal impact and anti-replicative effect, respectively (Fig. 6aI,bI). However, anti-HCV and anti-HSV1 standard drugs only demonstrated anti-replicative mode (78.98 ± 0.264% and 52.81 ± 1.093%, respectively). The anti-HSV action mode of biosurfactant was also supported by the morphology of treated Vero cells at three different protocols, illustrating that virucidal mode of biosurfactant and anti-replicative mode of acyclovir were able to maintain cells in healthy shaped compared to infected untreated cells (Fig. 6c). Also, the anti-replicative mode of biosurfactant was noticeable, but to a lesser extent than the same mode of acyclovir. Meanwhile, Vero cells treated by an adsorptive mode of biosurfactant or acyclovir lysed with no improvement over the untreated infected cells (Fig. 6c). For a more declaration of mechanisms-mediated antiviral activity of biosurfactant, its binding potential to viral envelope proteins and suppressive effect on the main viral replication enzymes (polymerases) were determined. After 2 h incubation, the binding percentages of biosurfactant to HCV E2 and HSV gp D were 94.15 ± 0.475% and 96.11 ± 0.198%, respectively, whereas reference drugs (sofosbuvir and acyclovir) did not exhibit this reactivity (Fig. 6aII,bII). Moreover, biosurfactant can inhibit the activity of HCV RNA polymerase (NS5B) and HSV DNA polymerase by 50% at 2.28 μg/mL and 4.387 μg/mL, respectively, compared to sofosbuvir and acyclovir which had lower IC_50_ values (1.70 μg/mL and 0.913 μg/mL, respectively) as illustrated in Fig. 6aIII,bIII.

### Molecular docking studies

#### Docking ethyl iso-allocholate to HCV E2 epitope

**Figure 7:**
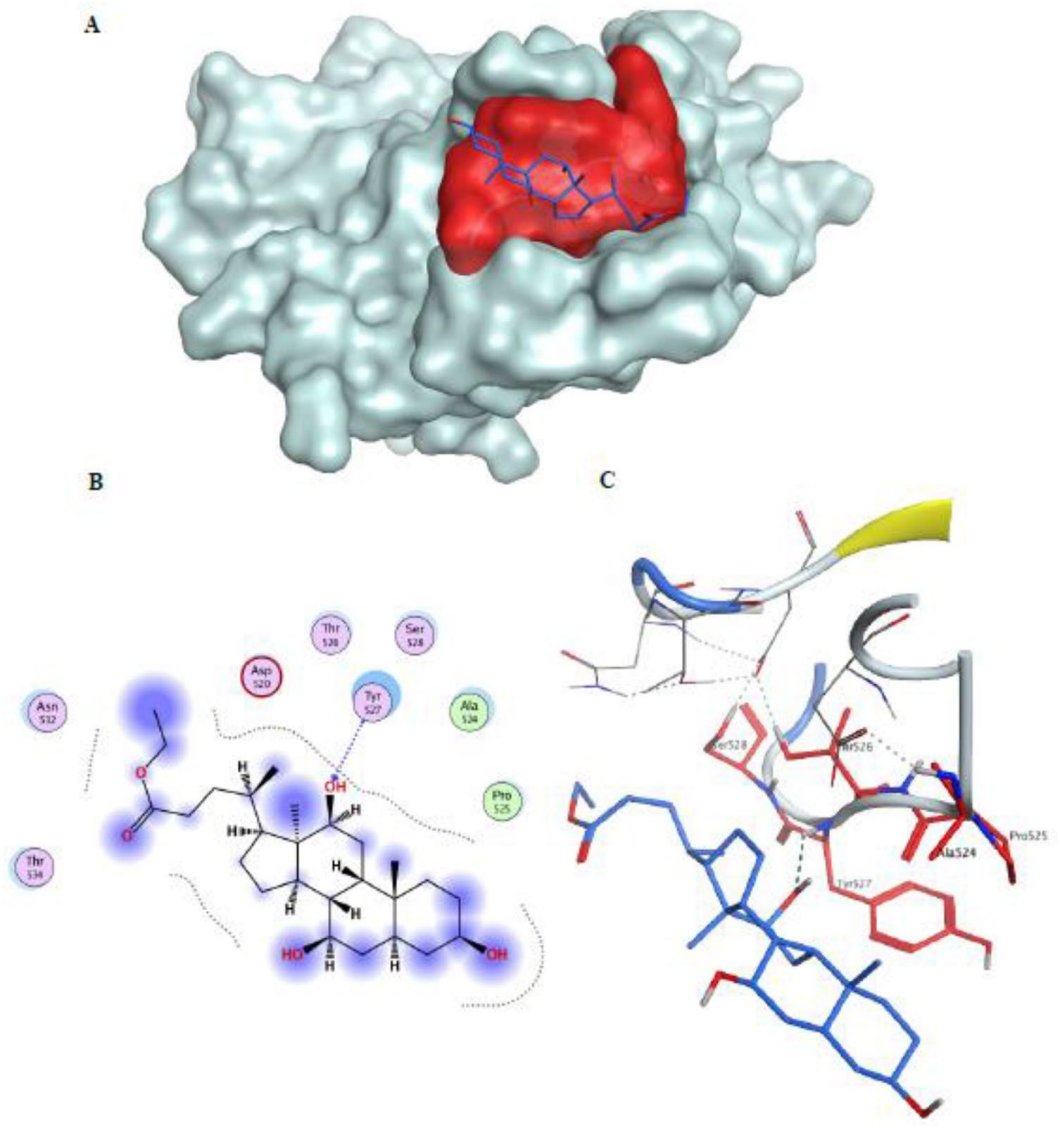
(**A**) Docking ethyl iso-allocholate (blue sticks) to epitope III (red molecular surface) on HCV E2 (white molecular surface), (**B**) 2D interactions, and (**C**) 3D binding mode of ethyl iso-allocholate (blue sticks) with epitope III amino acid residues ^524^APTYSW^529^ (red sticks) of the E2 ectodomain (PDB ID: 4 MW).

Herein, the epitope III residues were located on the HCV E2 protein (PDB ID: 4MWF) and identified as the receptor site after default structure preparation prior to docking ethyl iso-allocholate to the viral envelope employing Molecular Operating Environment (MOE) software package version MOE 2019.102. Flexible docking simulations (Fig. 7) showed that ethyl iso-allocholate resided well and was bound to the specified motif with low binding energies (ΔG = − 4.682 kcal/mol) through hydrogen bonding interaction involving the epitope’s key amino acid Tyr527.

#### Docking ethyl iso-allocholate to HSV gD

**Figure 8:**
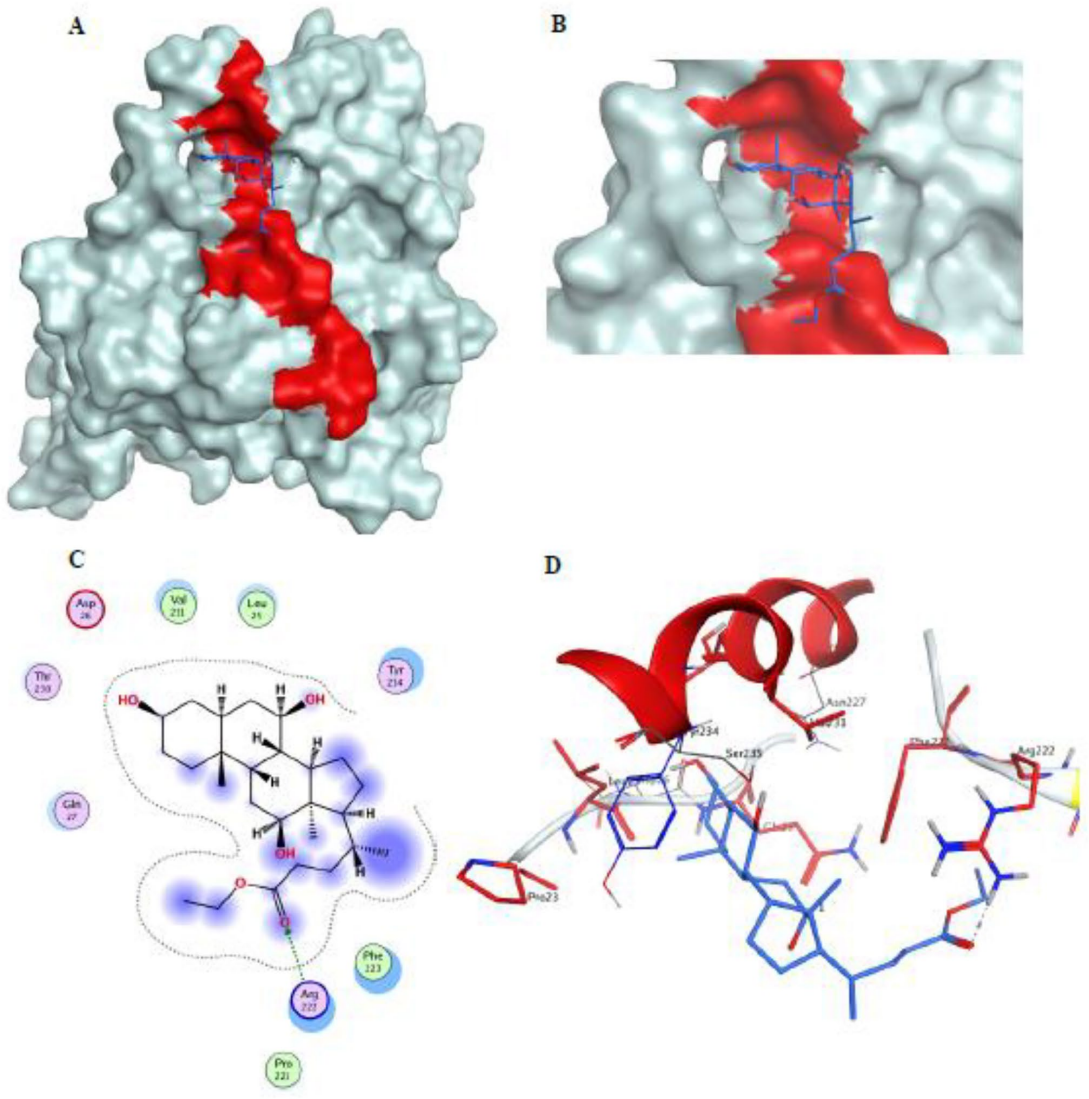
(**A**) Docking ethyl iso-allocholate (blue sticks) to the gD/nectin interaction site (red molecular surface) within the HSV gD (white molecular surface), (**B**) close-up view of ethyl iso-allocholate (blue sticks) contact sites on the gD motif involved in nectin binding, (**C**) 2D interactions, and (**D**) 3D binding mode of ethyl iso-allocholate (blue sticks) with key amino acids (red sticks) on gD (PDB ID: 3U82^38^).

In the current computational study, the structural coordinates of HSV gD/nectin-1 complex were retrieved from the Protein Data Bank (PDB ID: 3U82 ^19^). Unwanted residues were eliminated, then the structure was prepared utilizing MOE default protocol. gD was freed from nectin-1 and the aforementioned gD 21 residues were carefully mapped and assigned as the receptor for docking ethyl iso-allocholate. Flexible docking results (Fig. 8) demonstrated that ethyl iso-allocholate was able to bind the specified motif with acceptable binding affinity (ΔG = − 5.842 kcal/mol) displaying hydrogen bonding interactions with the gD key residue Arg222.

## Discussion

Halophilic archaea or haloarchaea are the most dominate in hypersaline environments. To survive under such extreme conditions, haloarchaea are adapted to function optimally in environments with high salt concentrations and, sometimes, with extreme pH and temperatures. El-Hamra Lake in Wadi El-Naturn was selected for the isolation of halophilic archaeal strains. Isolation of haloarchaea from Wadi El-Natrun was the interest of many researchers^22^. In the current work, biosurfactant production by the isolated archaea was tested qualitatively using different techniques including ST measurement, blood haemolysis, oil-spreading technique and emulsification index as described^23^. Two isolates (M6 and A5) were able to produce biosurfactant as indicated by a high EI%, and the reduction in the ST of cell-free supernatant techniques. One isolate M6 showing pigment and biosurfactant production was phenotypically characterized via morphological, molecularly identified as *Natrialba* sp., strain M6 (ac: MK063890), as mentioned previously by Hegazy et al.^21^, and ultimately was selected for its pronounced production of biosurfactant. Sequential optimization approaches were employed to maximize its production yields.

Different variables that assumed to affect the biosurfactant production were investigated using PBD^24^. Such statistical method was previously employed for the biosurfactant production by a marine *Bacillus* sp.^7^, and a halophilic archaeon *Natrialba* sp. C21^5^. Through, it was reported that *Natrialba* sp. C21 able to produce biosurfactant to facilitate the uptake of aromatic hydrocarbons and their biodegradation even at high salt concentration, the effect of using different carbon sources in the culture medium (glucose and glycerol) on the production of biosurfactant was investigated as well. Best to our knowledge, the type of carbon source is considered a vital limiting factor in microbial biosurfactant production process^25^. We found that glucose and ammonium nitrate were more effective when in negative level than pH and glycerol when in positive. Thus both glycerol and pH were selected for further optimization step, since both ammonium nitrate and glucose showed a negative effect on the biosurfactant production with a low value (− 1). Heryani and Putra, stated that a high glucose concentration caused an inhibition for the production of biosurfactant due to the formation of acidic metabolites^26^. BBD was applied, in the current study, to evaluate the effect of pH, glycerol, agitation, and sodium chloride as independent variables on the biosurfactant production and applied in order to identify the optimum response region for these factors as described by Box and Behnken^27^. The polynomial model was used to estimate the optimal levels of the four selected factors, which were found to be pH 12, 3% glycerol, 150 rpm agitation, and 20.8% NaCl. The highest biosurfactant concentration was obtained by applying the above mentioned parameters. The partially purified biosurfactant was characterized using GC–MS analysis as recommended by Jerković et al.^28^, and the data indicated that the recovered extracts contained a diverse group consists of 34 compounds, mostly biosurfactant as they are showing bipolar nature, hydrophobic hydrocarbon chain and charged function group also most of these compounds are fatty acids. In addition, total proteins, carbohydrates, and lipids analyses indicated that biosurfactant components are mainly lipid (41%) and protein (31%). Based on the biochemical and zetasizer results, the recovered biosurfactant extract from M6 strain might be cationic lipopeptide, also the FTIR indicated the absorption in the region from 1500 to 1650 cm^−1^ which not normally observed in the FTIR spectra of rhamnolipid biosurfactants, which differentiates the unique nature of LIP-BS from rhamnolipids. This finding is in agreement with Habib et al.^29^ who stated that the biosurfactant synthesised by *Rhodococcus* sp. ADL36 might be a lipopeptide as the relative content of the protein and lipid was approximately 25% and approximately 64%, respectively. Because of the negatively charged lipid bilayer of the cell membrane, the cationic property of biosurfactant aids in enhancing its uptake in viral host cells and subsequently, increasing biological activities (e.g., inhibition of viral replication-mediated antiviral activity).

The antiviral activity of the biosurfactant produced by *Natrialba* sp., M6 was studied against HCV 4a and HSV1, after detecting its safe dose and CC_50_ in normal host viral cells (> 8 μg/mL and 268 μg/mL, respectively). With high SI values, the qPCR results of neutralizing and anti-replicative effects of biosurfactant were supported with high percentages (94.15% and 96.11%) of biosurfactant reactivity against viral envelopes (HCV-E2 and HSV gpD, respectively) and low IC_50_ (2.28 µg/mL and 4.39 µg/mL) for inhibiting HCV RNA and HSV DNA polymerases, respectively. Its direct reactivity to viral envelopes resulted in viral elimination percentages of > 98%, which was higher than the inhibitory polymerases-mediated anti-replicative effect (37.41–42.12%). Our findings are consistent with previous studies which illustrated that biosurfactant from *Bacillus subtilis* disrupted HSV lipid membrane and partially capsid as mechanisms of viral inactivation^30^. Besides it inactivated envelope viruses of porcine epidemic diarrhea and transmissible gastroenteritis via inhibiting viral fusion to host cell membrane without demonstrating any inhibitory activity during the adsorption phase^31^. Also, the current study corroborates previous finding that lipopeptide (MA026) which was extracted from the fermentation broth of *Pseudomonas* sp. RtIB026, inhibited HCV entry in a dose-dependent manner^32^. A previous study demonstrated that anionic surfactant (sodium lauryl sulfate) denatured viral envelope proteins of HSV as well as HIV and Semliki Forest virus^33^. Furthermore, another previous study reported that sodium lauryl sulfate inhibited HSV1 infectivity after preincubation with HSV1, possibly due to viral GD binding, but not after pretreatment with Vero cells^34^. Regarding anti-replicative effect of biosurfactant (Fig. 6a,bIII), previous study found that 15 μg/mL surfactin of *Bacillus subtilis* suppressed completely RNA replication of envelope viruses^32^.

The HCV E2 protein has been recognized as the prime antibody target^35,36^ given its essential role in promoting viral entry into hepatocytes through various host cell entry factors, chief among them CD81^37,38^. Accordingly, most of the neutralizing antibodies target the CD81-binding loop on E2^38,39^ that is mapped to the residues between 523 and 540, of which the segment^524^APTYSW^529^ known as epitope III, represents the most important and highly conserved motif^40^, especially the residues Tyr527 and Trp529^39–41^. Also multiple surface envelope proteins are involved in the HSV entry and fusion. Among them, glycoprotein D (gD) has crucial role by binding to the host receptor nectin-1^42^, a member of the nectin and nectin-like molecules family belonging to immunoglobulin (Ig)-like cell adhesion molecules that mediate cell–cell adhesion and other regulatory functions^43,44^. Studies focused on elucidation of the molecular basis of gD/nectin-1 interaction for understanding the virus entry mechanism and introducing efficient anti-HSV agents. Hence, the structural basis of gD/nectin-1 binding has been resolved, and most importantly the key amino acid residues at the complex interface were characterized, where a total of 21 amino acids (including P23, L25, Q27, R36-H39, Q132, V214-F223, T230, V231 and Y234) were found within 4.5 Å distance from the nectin-1 entity. Of which, 7 belong to the *N*-terminal extension, 13 locate within the *C*-terminal extension and 1 residue is from the Ig core^45^. Herein, the epitope III residues were located on the HCV E2 protein (PDB ID: 4MWF^46^) and gD/nectin were identified as the receptor site for HCV and HSV after default structure preparation prior to docking ethyl iso-allocholate to the viral envelope employing Molecular Operating Environment (MOE) software package version MOE 2019.102^47^. Flexible docking simulations showed that ethyl iso-allocholate resided well and was bound to the specified motif with low binding energies (ΔG = − 4.682 kcal/mol) through hydrogen bonding interaction involving the epitope’s key amino acid Tyr527, and also demonstrated that ethyl iso-allocholate was able to bind the specified motif with acceptable binding affinity (ΔG = − 5.842 kcal/mol) displaying hydrogen bonding interactions with the gD key residue Arg222 According to our knowledge, no previous study investigated the antiviral activity of biosurfactant on HCV, including its E2 reactivity and inhibitory potential on replication enzyme. Based on our findings, such biosurfactant represents a potential novel candidate for suppressing the entry and replication of HCV and HSV, without causing cytotoxicity in host cells. It can be used as local treatment for viral infection and natural inhibitor of viral replication as well as preventer of viral transmission.

## Conclusion

For the first time, we describe the extraction of biosurfactant from a halophilic archaeon (*Natrialba* sp., M6), as well as the optimum conditions for maximum production, which were pH 12, 3% glycerol, 150 rpm agitation, and 20.8% NaCl. It has also been proven to have anti-HCV and anti-HSV1 activity. This novel study also unveiled its antiviral mechanisms (reactivity against HCV E2 and gpD as well as inhibitory potency on HCV NS5B and HSV polymerase) which led to halting entry and replication of both viruses. In contrast to standard drugs’ single antiviral mode (anti-replicative), biosurfactant exhibited high direct virucidal activity as well as anti-replicative activity to a lesser extent. Therefore, such biosurfactant could be addressed as a natural effective antiviral agent and could be tested against many other viruses as a future prospective.

## Materials and methods

### Isolation source and screening for biosurfactant production

Samples were collected from El-Hamra Lake, Wadi El-Natrunt, where a particular isolation protocol for halophilic archaea was followed using a specified culture medium (g/L): casamino acids, 5; KH_2_PO4, 1; MgSO4·7H2O, 0.2; NaCl, 200; trace metals, 1 mL; and Na_2_CO3, 18. The trace metal solution contained (g/L) ZnSO4·7H2O, 0.1; MnCl_2_·4H2O, 0.03; H3BO3, 0.3; CoCl_2_·6H2O, 0.2; CuCl_2_·2H2O, 0.01; NiCl_2_·6H2O, 0.02; and Na_2_MoO4·H2O, 0.03 as described by Hegazy et al.^21,48^. The chemical analysis of water and sediments for El-Hamra Lake, Wadi El-Natrun, was carried out at the central lab, City of Scientific Research and Technological Applications, Borg El-Arab, Egypt^19^. Metal concentrations were measured using atomic absorption spectroscopy (Analytical Jena AG, 07745 Jena, Germany). Samples were diluted prior to analysis to set within the calibration linear range, where blank and standards solutions for device calibration were used.The chemical parameters of water sample including silica, phosphate, chloride, sulphate, bicarbonate and total nitrogen were carried out using standard methods. For testing the ability of obtained isolates for biosurfactant production; seed culture was prepared from the tested isolates until OD ~ 0.9. The standard inoculum of 1 mL was used to inoculate 250 mL flasks each containing 100 mL basal medium adjusted to pH 11. Flasks were shaken at 200 rpm and incubated at 37 °C for 7 days before being tested for biosurfactant production in cell free supernatant using the following methods.

### Oil spreading technique

Two drops of crude gas oil (a petroleum distillate product) were placed on the surface of distilled water in a petri dish (150 mm in diameter). Then, 10 µL of the free cells culture supernatant were gently put on the center of the oil film. The formation of clear area indicated the presence of biosurfactant and scored positive result, while absence of this clear area was scored as negative result^49^. In all applied techniques for biosurfactant detection a medium without culture was tested as − ve control while a chemical surfactant like Tween 20 at 1% is observed as + ve control.

### Surface tension measurement

ST of isolates supernatants were measured as mentioned by Haba et al.^50^ isolates cultures were centrifuged at 15,000 rpm for 15 min and the ST of the supernatants was measured using a tensiometer (TDI, Lauda, Germany) and expressed as mN/m using distilled water as a reference.

### Emulsification index (% EI_24_)

Emulsification activity of isolates was measured using the method described by Iqbal et al.^51^. 4 mL of petroleum gas oil-distillate were added to 4 mL of the culture supernatant in a test tube, and vortexed at high speed for 5 min. After 24 h the emulsion stability was determined, and the emulsification index (% EI_24_) was calculated by dividing the emulsion layer measured height by the mixture total height and multiplying by 100.

### Haemolytic activity

Haemolytic activity was tested by screening the isolates on blood agar plates containing 10% (v/v) sheep blood and incubated at 37 °C for 24 h. Haemolytic activity was detected by the presence of clear zone around colonies which is indicative of surfactant production^23^.

### *Natrialba* sp. M6 phylogeny

The sequence of 16s *rRNA* (MK063890) from *Natrialba* sp. encoded M6 (The best screened isolate for biosurfactant production) was used for phylogeny creation. BioEdit Sequence Alignment Program^52^ was applied for comparative sequence analyses through ClustalW modulus of the BioEdit program then a phylogenetic tree was constructed using MEGA software version 4.0.2^53^.

### Surfactant optimization using PBD

PB experimental design^24^ was used to evaluate the relative significance of 14 culture factors, including medium components and other physical parameters on the biosurfactant production by the haloalkaliphilic archaeon *Natrialba* sp. M6. Based on the PB factorial design, each factor was tested at 2 levels: ʻ− 1ʼ for the low level, and ʻ+ 1ʼ for the high level. In addition, the matrix design of the tested factors was screened in 16 experimental trials. All trials were done in 250 mL flasks containing 100 mL of the medium. The response was the ST measured in mN/m using a tensiometer, then expressed as the reciprocal of the corrected ST × 1000.

PBD is based on a first order model: Y = β0 + ∑ βi xi.

A pre-optimization step should be done for subsequent optimization step. In this step, a pre-optimization formula was prepared, where the most significant factors were fixed at their optimum levels which obtained from PBD. On the other hand, the other variables with a negative effect value were fixed at their ʻ− 1ʼ coded values, and those with a positive effect value were fixed at their ʻ+ 1ʼ coded values. The purpose of this step is to confirm the results of PBD and to create the essential formula for further optimization step.

### Response surface methodology

The most significant variables were selected for further optimization experiment of their optimal level with respect to reciprocal of corrected ST as a response expressing the biosurfactant yield. The four significant variables were pH (X_1_), glycerol (X_2_), agitation (X_3_) and NaCl (X_4_). The low, middle and high levels of each variable were designated as − 1, 0 and + 1, respectively.

The equation for the four factors for biosurfactant was as follows:

$${\text{Y}} = \upbeta_{0} + \upbeta_{{1}} \left( {{\text{X}}_{{1}} } \right) \, + \upbeta_{{2}} \left( {{\text{X}}_{{2}} } \right) \, + \upbeta_{{3}} \left( {{\text{X}}_{{3}} } \right) \, + \upbeta_{{4}} \left( {{\text{X}}_{{4}} } \right) \, + \upbeta_{{{12}}} \left( {{\text{X}}_{{1}} {\text{X}}_{{2}} } \right) \, + \upbeta_{{{13}}} \left( {{\text{X}}_{{1}} {\text{X}}_{{3}} } \right) \, + \upbeta_{{{14}}} \left( {{\text{X}}_{{1}} {\text{X}}_{{4}} } \right) \upbeta_{{{23}}} \left( {{\text{X}}_{{2}} {\text{X}}_{{3}} } \right) \, + \upbeta_{{{24}}} \left( {{\text{X}}_{{2}} {\text{X}}_{{4}} } \right) \, + \upbeta_{{{34}}} \left( {{\text{X}}_{{3}} {\text{X}}_{{4}} } \right) \, + \upbeta_{{{11}}} ({\text{X}}_{{1}} )^{{2}} + \upbeta_{{{22}}} \left( {{\text{X}}_{{2}} } \right)^{{2}} + \upbeta_{{{33}}} \left( {{\text{X}}_{{3}} } \right)^{{2}} + \upbeta_{{{44}}} \left( {{\text{X}}_{{4}} } \right)^{{2}} ,$$

where, Y is the predicted response (biosurfactant), β_0_ is constant, β_1_, β_2_, β_3_ and β_4_ are linear coefficients, β_12_, β_13_ and β_23_ are cross product coefficients, and β_11_, β_22,_ β_33_ and β_44_ are quadratic coefficients^27,54^. Variables maximal predicted response and coefficients calculations were carried out using Microsoft Excel 2007.

### Recovery of the biosurfactant

The biosurfactant of *Natrialba* sp. M6 was collected by centrifugation (15,000 rpm, 30 min) from the culture supernatant. For biosurfactant extraction, the cell-free culture supernatant was acidified to pH 2 using conc HCl and then kept at 4 °C overnight. The precipitate was harvested by centrifugation (15,000 rpm, 30 min). The biosurfactant residue after precipitation was dried and weighed and dissolved in known volume of 0.1 M sodium bicarbonate^55^.

### Protein, lipid and carbohydrate quantification

Protein, lipid and carbohydrate contents of the recovered biosurfactant produced by *Natrialba* sp., M6 were determined colorimetrically. Total protein contents was measured using 2.5 mL of alkaline copper solution were added to500 µL of biosurfactant, standards and blank, mixed well and allowed to stand for 10 min or more at room temperature. Then, 250 µL of diluted Folin reagent was added rapidly and mixed within one second or two. After 20 min, the samples were read at 750 nm wavelength and calculated from a standard curve which prepared using different concentrations of human serum ranged from 100 to 500 µg/mL^56^. Total lipid content was also measured using test tubes containing 500 µL of biosurfactant, standards and blank, 250 µL of concentrated sulfuric acid were added to them and mixed well. Test tubes were placed in boiling water for about 5 min, then 5 mL of the phospho-vanillin reagent were added to each tube, mixed well, and incubated at 37 °C in water bath for 15 min. The tubes were cooled for about 5 min and then within 30 min, the absorbance was measured at wave 540 nm wavelength. Standards were prepared from cholesterol standard with different concentrations ranged from 50 to 200 mg/dL^57^. Carbohydrate content was determined using 600 µL of biosurfactant sample, standards and blank, 600 µL of phenol (5%w/v) were added and mixed well with 3 mL concentrated sulfuric acid. The test tubes were left at room temperature for 30 min then measured at 490 nm wavelength^58^. Standards series were prepared from D-glucose with concentrations 20–100 mg/L. All these measurements were carried using a double beam meter UV/Vis spectrophotomer SP-8001 at the marine chemistry lab, National Institute of Oceanography and Fisheries, Alexandria, Egypt.

### Elemental analysis (EA)

EA including N, C, H and S of the recovered biosurfactant produced by *Natrialba* sp., M6 were carried out at the central lab, City of Scientific Research and Technological Applications, Borg El-Arab, Egypt using Elemetar Analysensysteme GmbH, Germany.

### Gas chromatography–mass spectrometry (GC–MS) analysis

GC–MS analysis was performed according to Jerkovic et al.^28^. Using an Agilent technologies (GC) equipped with mass selective detector (MS), HP-5MS at the marine pollution lab, National Institute of Oceanography and Fisheries, Alexandria, Egypt, The constituents were identified by comparison of their mass spectral data with those standard compounds from NIST (National Institute of Standards and Technology) Spectral Library.

### Fourier transform infrared (FTIR) spectroscopy analysis

The chemical structure of the dried produced biosurfactant was partially identified using a band Find-Memory-27 spectrophotometer. A mixture of approximately 1 mg of the tested material and 300 mg of pure dry potassium bromide (KBr) was pressed into discs. The measurements obtained infrared spectra between 400 and 4000 cm^−1^.

### Investigation of anti-HCV activity of biosurfactant

#### Cell culture

Human PBMCs (host HCV cells) were isolated from the collected heparinized blood of healthy volunteers and HCV-infected serum samples were separated from the collected blood of HCV patients for investigating cytotoxicity and antiviral activity of biosurfactant. All these experiments were approved (authorization number: 0305142, by Human Research Ethical Committee (REC) of Faculty of Medicine (Alexandria University), Egypt. All experimental protocols were conducted in accordance with guidelines of National Health and Medical Research Council policies as well as Egyptian Ministry of Health and Population. The written informed consent was obtained from all participants, including healthy volunteers and HCV patients.

Human PBMCs, as HCV host cells, were isolated by Ficoll-Hypaque density gradient centrifugation method as described previously^59^. In a brief, human blood samples were precisely placed on Ficoll-Hypaque (StemCell Technologies, Canada) and centrifuged for 30 min at 2000 rpm. The interface PBMC layer was then collected and centrifuged twice for 10 min at 1650 rpm. The cell pellet was suspended in 10% fetal bovine serum (FBS)-containing Roswell Park Memorial Institute (RPMI)-1640 medium. Using the trypan blue exclusion method, the viability and counting of the obtained PBMCs were determined. Regarding HSV host cells, green monkey kidney epithelial (Vero) cells were cultured in Dulbecco’s modified Eagle medium (DMEM) supplemented with 10% FBS. Ten-fold dilutions of acyclovir sensitive-HSV1 (KOS) were incubated with monolayer of Vero cells (90% confluent), respectively, in 96 well plates. After 2 h in 5% CO_2_ incubator (New Brunswick Scientific, Netherlands), unabsorbed viruses were aspirated then DMEM/10% FBS was added. Then plates were incubated in 5% CO_2_ incubator for 3 days. After that, 20 µL of 5 mg/mL MTT solution (Sigma, USA) was added to each well and the plates were incubated at 37 °C for 4 h. Following the removal of MTT, DMSO was added and the absorbance was measured at 570 nm using an ELISA reader (BMG LabTech, Germany). MTT assay^60^ was used to determine %cell viability in all infected wells comparing with wells of uninfected healthy cells to calculate the tissue culture infectious dose (TCID50) using the formula of Reed and Muench method^61^.

#### MTT assay for investigation of biosurfactant cytotoxicity on viral host cell lines

The cytotoxicity of biosurfactant on PBMCs and Vero cells was performed using MTT assay^53^. After 72 h incubation of its serial concentration with these viral host cells, in 5% CO_2_ incubator, MTT assay was carried out as described above. The percentage of cell viability was calculated in order to estimate the safe concentration (EC_100_) and cytotoxic concentration (CC_50_) at 100% and 50% cell viability, respectively.

### Determination of effective concentration (EC_50_) for anti-HCV and anti-HSV1 activity of biosurfactant

The virus inoculum (at 100 TCID50/mL) was added to 96 well plates containing monolayer of Vero cells and incubated for 2 h at 37 °C in 5% CO_2_ incubator. After that, the unabsorbed viruses were aspirated and replaced with fresh culture medium containing successive concentrations of biosurfactant. Then cells were incubated for 3 days in 5% CO_2_ incubator. All wells (healthy, infected-untreated and treated infected cells) were stained with MTT, as described above, to estimate % reduction in the lysis of infected cell following exposure to biosurfactant.

Regarding detection of anti-HCV efficacy, 1 × 10^6^ cells human PBMCs were incubated with 2.9 × 10^4^ HCV (excluding negative control wells) in 6-well culture plate. After overnight incubation in CO_2_ incubator at 37 °C, the infected medium was replaced with a fresh medium containing 10% FBS (for positive control wells) or serial concentration of biosurfactant or sofosbuvir. Following 72 h in CO_2_ incubator, the viral elimination (%) was quantified using TaqMan-based real time PCR as described below.

The dose (EC_50_) values at which 50% HCV clearance and 50% inhibition of HSV1-mediated cell lysis were calculated by the Graphpad Instat software. Furthermore, selectivity index (SI) was estimated as the ratio of CC_50_ to EC_50_.

### Quantitative PCR analysis of anti-HCV and anti-HSV1 activities declaring its action mode

The viral host cells, human PBMCs (1 × 10^6^ cells/well) and Vero (0.6 × 10^6^ cells/well) were seeded, separately, in 6-well culture plate. All wells, except negative control wells, were overnight and 2 h incubated with 2.9 × 10^4^ HCV (genotype 4a) and 10^–4^ HSV1, respectively, in CO_2_ incubator at 37 °C, 5% CO_2_ and 95% humidity. Then the infected medium was replaced with a fresh medium containing 10% FBS for positive control wells. For evaluating the anti-replicative potential, the infected medium was exchanged with medium containing 10% FBS and EC_100_ of biosurfactant or reference antiviral drug (sofosbuvir and acyclovir, respectively) and incubated for 72 h in CO_2_ incubator. For investigating the neutralization (direct virucidal) effect of biosurfactant, it (at EC_100_) was incubated for 2 h, in 5% CO_2_ incubator, with viruses, before being incubated with 2 h with host cells. Meanwhile, the blocking effect on viral entry was assessed by pretreating host cells with biosurfactant (at EC_100_) for 2 h, then discarding and adding viruses to their host cells for another 2 h. All these experiments, the untreated and treated infected cells were harvested, after 72 h, for determination of viral load using qPCR.

The fully automated Cobas AmpliPrep apparatus with Cobas TaqMan analyzer (Roche Diagnostics, USA) was used to quantify HCV-RNA in both untreated and treated HCV-infected PBMCs PBMCs with using fluorescent labelled oligonucleotide probes by following the manufacturer’s instructions of Abbott real time HCV kit (Abbott Molecular Inc, USA).

In the case of HSV1, viral DNA was extracted from the untreated and treated wells using Qiagen extraction kit then TaqMan-based real time PCRs were carried out according to Kessler et al.^62^. The used HSV primers were (5ʹ-CATCACCGACCCGGAGAGGGAC-3ʹ and 5ʹ-GGGCCAGGCGCTTGTTGGTGTA-3ʹ) with probe 5ʹ-6-carboxyfluorescein (FAM)-CCGCCGAACTGAGCAGACACCCGCGC-6-carboxytetramethylrhodamine (TAMRA).

### ELISA assessment of biosurfactant reactivity to envelope proteins of HCV and HSV

Briefly, MaxiSorp 96 well plate was coated with HCV E2 (2 μg/mL, R&D, Minneapolis, USA) or HSV gp D (1 μg/mL). After overnight incubation at 4 °C, coated wells were washed, blocked with 5% skimmed dry milk for 2 h and washed again. The serial concentrations of biosurfactant were added. After 2 h, plate was washed and incubated with anti-HCV E2 antibody or anti-HSV gp D antibody (Antibodies-online GmbH, Germany) for 1 h then unbound antibody was discarded and alkaline peroxidase-conjugated secondary antibody (Abcam, UK) was added. Next 1 h, wells were washed to remove unbinding mixture and 3,3ʹ,5,5ʹ tetramethylbenzidine (substrate)—(Sigma, USA).

### Inhibitory effect of biosurfactant on HCV and HSV polymerases activity

The inhibitory potential of biosurfactant on HCV NS5B (Creative biolabs, USA) polymerase activity was performed using the modified method of Bellecave et al.^63^. Briefly, serial concentrations of biosurfactant were added to a mixture reaction of 150 nM NS5B, 86 nM RNA template, 1 mM dithiothreitol (DTT), 5 mM MgCl_2_, 40 mM NaCl, 20 mM Tris pH 7.5 and 0.5 mM each of nucleotides including 10 µCi radiolabeled [^32^P-UTP, PerkinElmer LAS, UK]. After 2 h, the newly synthesized DNA was then transferred to the filter paper disks and precipitated with trichloroacetic acid before being measured with a scintillation counter. For DNA polymerase of HSV, serial concentrations of biosurfactant and such enzyme were incubated with reaction mixture of 8 mM MgCl_2_, 0.5 mM DTT, 50 mM Tris–HCl pH 8, 100 mM ammonium sulfate, 0.5 μg/mL albumin and 100 μM deoxynucleotides with 1 µCi radiolabeled [^3^H-dTTP, PerkinElmer LAS, UK]. After 1 h incubation at 37 °C, reaction was terminated by acid precipitation then radioactivity of newly synthesized DNA was measured using scintillation counter^64,65^.

### Molecular docking studies

#### Structures acquisition and preparation

The available three-dimensional crystal structures of hepatitis C virus envelope glycoprotein E2 (HCV E2), herpes simplex virus glycoprotein D (HSV gD) and surfactin were retrieved from the Protein Data Bank (PDB, www.rcsb.org) PDB IDs: 4MWF^46^, 3U82^45^ and 2NPV^66^, respectively for docking employing Molecular Operating Environment (MOE) software package version MOE 2019.102, Chemical Computing Group, Montreal, Canada^47^. Unwanted residues and ligands were eliminated. Ethyl iso-allocholate was built in silico. The structures were prepared and refined employing the default “Structure preparation” MOE setting, then energy minimized employing Amber10: EHT force field with reaction-field electrostatics (an interior dielectric constant of 1 and an exterior dielectric of 80) using an 8–10 Å cutoff distance.

### Docking simulations

The key amino acid residues in HCV E2 and HSV gD were located and identified as the receptor sites. Ethyl iso-allocholate was docked to the selected motifs applying the Triangular matcher algorithm and Alpha HB as placement and scoring functions generating the top 10 non-redundant poses of the lowest binding energy conformers. Docking was conducted with induced fitting protocol. Results were recorded as S-scores with RMSD value < 2.5 Å. Graphical representations of the molecular interactions were generated and inspected.

## Supplementary Information

Supplementary Figure 1.

## Data Availability

All data produced during this study are included in this published article.
