# Supplementary material for: Bioprocess development for biosurfactant production by Natrialba sp. M6 with effective direct virucidal and anti-replicative potential against HCV and HSV
Source: Sci Rep. 2022 Oct 4;12:16577. doi: 10.1038/s41598-022-20091-0 (PMC9531635; doi:10.1038/s41598-022-20091-0)
Supplement: Supplementary file 1 — Supplementary Figure 1. [file 41598_2022_20091_MOESM1_ESM.docx]

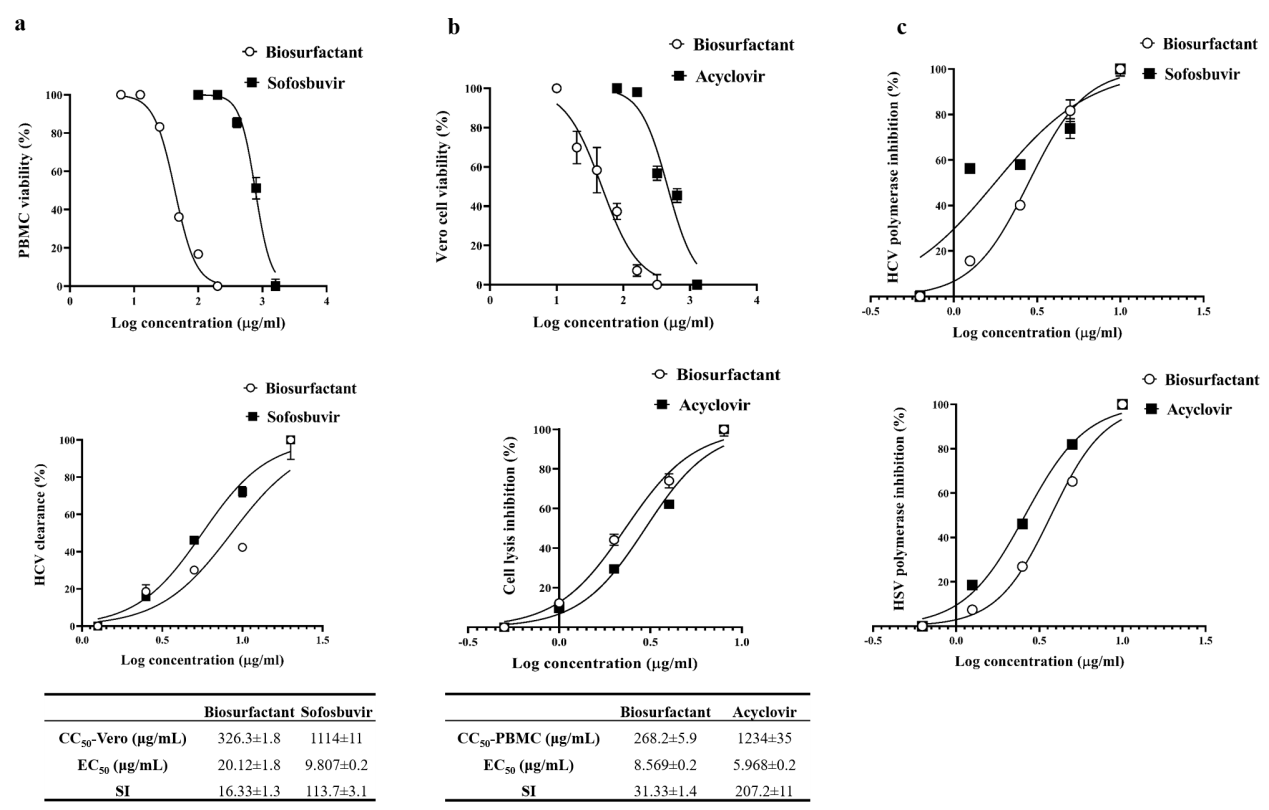


**Supplementary Figure 1. Dose response curves of cell viability, antiviral and polymerase inhibition. (a)** Peripheral blood mononuclear cell (PBMC) viability (%) and qPCR results of HCV clearance (%) after treatment with serial concentrations of biosurfactant and sofosbuvir with the summarized table of cytotoxic concentration (CC_50_) at 50% viral host cell viability, effective concentration (EC_50_) at 50% viral clearance and the calculated selectivity index of antiviral effect (SI). (**b**) Vero cell viability (%) and HSV1 clearance, in the term of cell lysis inhibition, upon the treatment with serial concentrations of biosurfactant and acyclovir with summarized table of CC_50_, EC_50_, and SI. (**c**) The percentage inhibition of HCV polymerase and HSV polymerase at serial dilutions of biosurfactant and standard antiviral drugs (sofosbuvir and acyclovir, respectively). All data from triple-repeated experiments (n=3) are expressed as mean±SEM.
